# Supplementary material for: Organelle scaling over a 100-fold cell size range
Source: bioRxiv. 2026 May 13:2026.05.13.724986. Preprint. [Version 1] doi: 10.64898/2026.05.13.724986 (PMC13192762; doi:10.64898/2026.05.13.724986)
Supplement: Supplement 5 [file NIHPP2026.05.13.724986v1-supplement-5.pdf]

bioRxiv preprint doi: https://doi.org/10.64898/2026.05.13.724986; this version posted May 13, 2026. The copyright holder for this preprint (which was not certified by peer review) is the author/funder, who has granted bioRxiv a license to display the preprint in perpetuity. It is made available under a CC-BY 4.0 International license.

SUPPLEMENTAL FIGURE CAPTIONS

Supplemental Figure 1: **ER content scales linearly with cell volume when Elo3-GFP is used to visualize the ER**. (**A**) Log-log plot of total ER content (Elo3-GFP signal) and cell volume. The best fit line, slope, and R^2 values are shown (n = 179 cells). (**B**) ER density for small (<100 μm^3) and large cells (>400 μm^3). Mean and standard deviation are shown. p = 0.221 by student's t-test (n = 38 small and 70 large cells). (**C**) Maximum intensity projection of a confocal Z-series (top) and single medial plane image (bottom) of cells of different sizes expressing the ER marker Elo3-GFP (cyan) and cytosol marker 3xmCherry (magenta) (DLY27372). The central slice is shown in greyscale to highlight fine ER structures. Dashed boxes indicate insets shown in D. Scale bar, 5 μm. (**D**) Top: Example confocal single glancing slice images of cortical ER from the regions in the dashed boxes in C. Bottom: mean cortical ER intensities measured from glancing slices in small (<200 μm^2) and large (>600 μm^2) cells. The mean and standard deviation are shown. p > 0.05 by student's t-test (n = 38 small and 70 large cells). Scale bar, 2 μm. (**E**) Maximum intensity projection of a confocal time series of a cell expressing ER marker Elo3-GFP (greyscale) and cytosol marker 3xmCherry (not shown) (DLY27372). Scale bar, 5 μm. (**F**) Quantification of cell volume (left), total ER content (middle), and ER density (right) for three representative cells. The top row is measured from the same cell shown in E. Red arrowheads indicate the times when mitosis took place. Grey boxes indicate the budded intervals.

Supplemental Figure 2: **Total Atg42-3xmNG signal is correlated with vacuole volume.** Plot of total Atg42-3xmNG signal and total vacuole volume per cell measured from cells expressing the vacuole lumen marker, Atg42-3xmNG, and cytosol marker, 3xmCherry (DLY25963). The best fit line, slope, and R^2 values are shown (n = 375 cells).

SUPPLEMENTAL VIDEO CAPTIONS

Video 1: Mitochondrial content increases as cells grow larger. Maximum intensity projection of a confocal time series of a cell switching between budding and nuclear cycles expressing the mitochondrial marker Cit1-GFP (cyan) and cytosol marker 3xmCherry (magenta) (DLY25312). This is the same cell analyzed in Figure 2E of the main text. Images were acquired every 10 min. Scale bar, 5 μm.

bioRxiv preprint doi: https://doi.org/10.64898/2026.05.13.724986; this version posted May 13, 2026. The copyright holder for this preprint (which was not certified by peer review) is the author/funder, who has granted bioRxiv a license to display the preprint in perpetuity. It is made available under a CC-BY 4.0 International license.

Video 2: ER content increases as cells grow larger. Maximum intensity projection of a confocal time series of a cell going through two nuclear cycles expressing the ER marker Sec61-GFP (cyan) and cytosol marker 3xmCherry (magenta) (DLY24944). This is the same cell analyzed in Figure 4A of the main text. Images were acquired every 10 min. Scale bar, 5 μm.

Video 3: Vacuole content increases as cells grow larger. Maximum intensity projection of a confocal time series of a cell switching between budding and nuclear cycles expressing the vacuole membrane marker Vph1-GFP (cyan) and cytosol marker 3xmCherry (magenta) (DLY24947). This is the same cell analyzed in Figure 5E of the main text. Images were acquired every 10 min. Scale bar, 5 μm.

Video 4: Peroxisome content increases as cells grow larger. Maximum intensity projection of a confocal time series of a cell switching between budding and nuclear cycles expressing the peroxisome marker Pex3-GFP (cyan) and cytosol marker 3xmCherry (magenta) (DLY25001). This is the same cell analyzed in Figure 7E of the main text. Images were acquired every 10 min. Scale bar, 5 μm.

bioRxiv preprint doi: https://doi.org/10.64898/2026.05.13.724986; this version posted May 13, 2026. The copyright holder for this preprint (which was not certified by peer review) is the author/funder, who has granted bioRxiv a license to display the preprint in perpetuity. It is made available under a CC-BY 4.0 International license.

Supplemental Figure 1

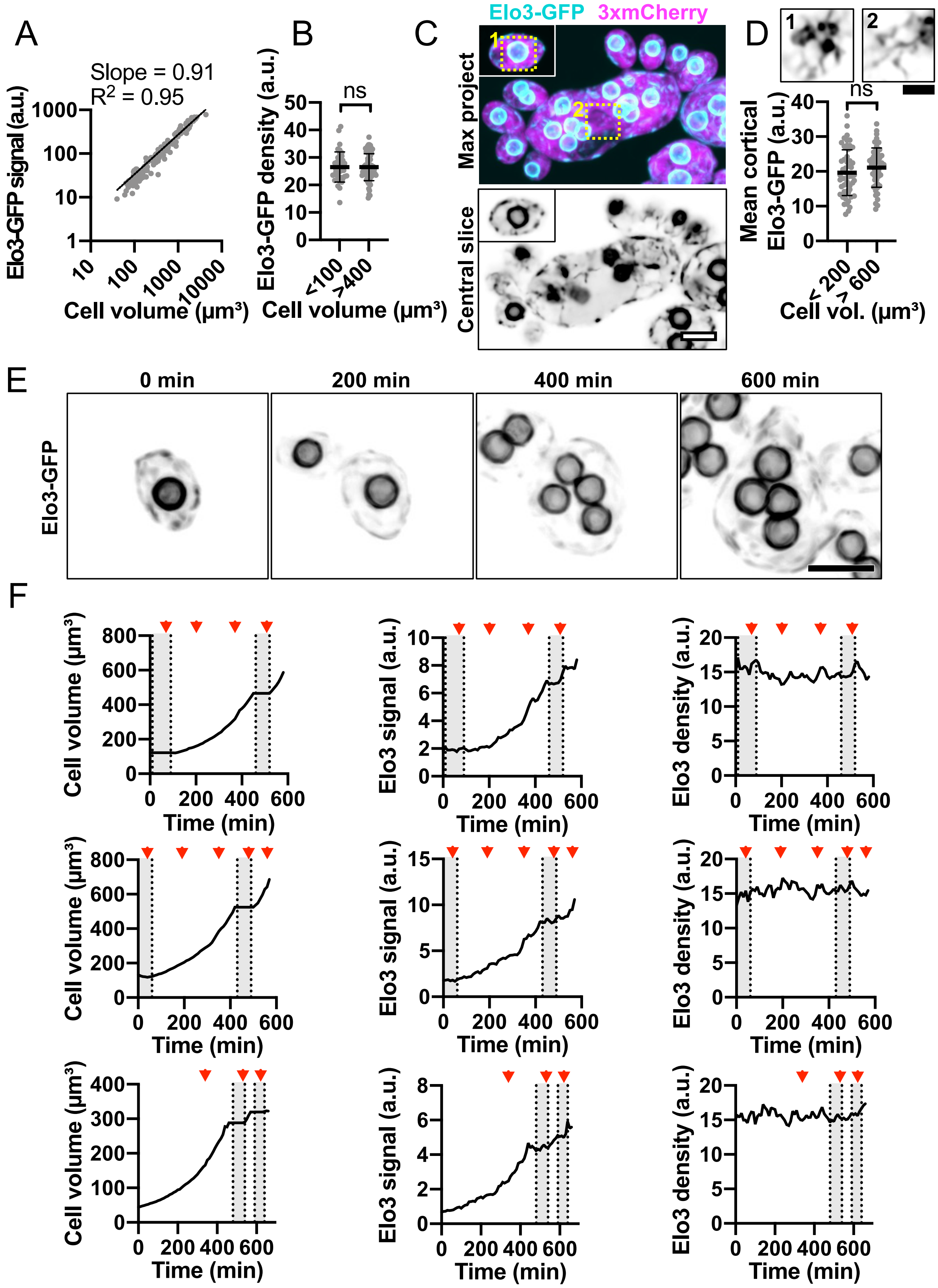

bioRxiv preprint doi: https://doi.org/10.64898/2026.05.13.724986; this version posted May 13, 2026. The copyright holder for this preprint (which was not certified by peer review) is the author/funder, who has granted bioRxiv a license to display the preprint in perpetuity. It is made available under a CC-BY 4.0 International license.

Supplemental Figure 1: **ER content scales linearly with cell volume when Elo3-GFP is used to visualize the ER**. (**A**) Log-log plot of total ER content (Elo3-GFP signal) and cell volume. The best fit line, slope, and R^2 values are shown (n = 179 cells). (**B**) ER density for small (<100 μm^3) and large cells (>400 μm^3). Mean and standard deviation are shown. p = 0.221 by student's t-test (n = 38 small and 70 large cells). (**C**) Maximum intensity projection of a confocal Z-series (top) and single medial plane image (bottom) of cells of different sizes expressing the ER marker Elo3-GFP (cyan) and cytosol marker 3xmCherry (magenta) (DLY27372). The central slice is shown in greyscale to highlight fine ER structures. Dashed boxes indicate insets shown in D. Scale bar, 5 μm. (**D**) Top: Example confocal single glancing slice images of cortical ER from the regions in the dashed boxes in C. Bottom: mean cortical ER intensities measured from glancing slices in small (<200 μm^2) and large (>600 μm^2) cells. The mean and standard deviation are shown. p > 0.05 by student's t-test (n = 38 small and 70 large cells). Scale bar, 2 μm. (**E**) Maximum intensity projection of a confocal time series of a cell expressing ER marker Elo3-GFP (greyscale) and cytosol marker 3xmCherry (not shown) (DLY27372). Scale bar, 5 μm. (**F**) Quantification of cell volume (left), total ER content (middle), and ER density (right) for three representative cells. The top row is measured from the same cell shown in E. Red arrowheads indicate the times when mitosis took place. Grey boxes indicate the budded intervals.

bioRxiv preprint doi: https://doi.org/10.64898/2026.05.13.724986; this version posted May 13, 2026. The copyright holder for this preprint (which was not certified by peer review) is the author/funder, who has granted bioRxiv a license to display the preprint in perpetuity. It is made available under a CC-BY 4.0 International license.

Supplemental Figure 2

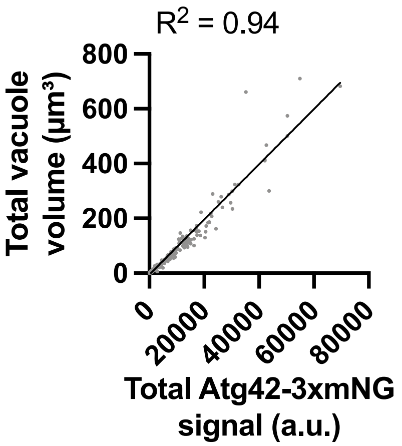

Supplemental Figure 2: **Total Atg42-3xmNG signal is correlated with vacuole volume.** Plot of total Atg42-3xmNG signal and total vacuole volume per cell measured from cells expressing the vacuole lumen marker, Atg42-3xmNG, and cytosol marker, 3xmCherry (DLY25963). The best fit line, slope, and R^2 values are shown (n = 375 cells).

bioRxiv preprint doi: https://doi.org/10.64898/2026.05.13.724986; this version posted May 13, 2026. The copyright holder for this preprint (which was not certified by peer review) is the author/funder, who has granted bioRxiv a license to display the preprint in perpetuity. It is made available under a CC-BY 4.0 International license.

Supplemental Table 1

Strain Name	Relevant Genotype	Source
DLY24944	*URA3:3xmCherry; SEC61-GFP:HYG* ^{R}	Wirshing et al., 2026
DLY24947	*URA3:3xmCherry; VPH1-GFP:HYG* ^{R}	Wirshing et al., 2026
DLY25001	*URA3:3xmCherry; PEX3-GFP:HYG* ^{R}	Wirshing et al., 2026
DLY25312	*URA3:3xmCherry; CIT1-GFP:NAT* ^{R}	Wirshing et al., 2026
DLY25963	*URA3:3xmCherry; ATG42-3xmNG:HYG* ^{R}	Wirshing et al., 2026
DLY27372	*URA3:3xmCherry; ELO3-GFP:NAT* ^{R}	Wirshing et al., 2026
DLY27440	*SEC61-3xmCherry:HYG* ^{R}*; Elo3-GFP:NAT* ^{R}	This study
